# UBC9: a novel therapeutic target in papillary thyroid carcinoma

**DOI:** 10.1007/s40618-024-02523-y

**Published:** 2025-03-01

**Authors:** Hui Zhang, Jingjing Wu, Huaiyuan Hu, Heng Tang, Kemeng Tan, Mengxue Hu, Genbao Zhu

**Affiliations:** 1 Department of pathology, Anhui Wanbei Coal-Electricity Group General Hospital, Suzhou, 234000 China; 2 International Joint Research Center of National Animal Immunology, College of Veterinary Medicine, Henan Agricultural University, Zhengzhou, 450046 China; 3 Longhu Laboratory of Advanced Immunology, Zhengzhou, 450046 China; 4 General Clinical Research Center, Anhui Wanbei Coal-Electricity Group General Hospital, Suzhou, 234000 China

## Abstract

**Background:**

Papillary thyroid carcinoma (PTC) is the most common type of thyroid cancer. Despite the favorable prognosis in some patients, there remains a risk of lymph node metastasis and death in some patients. Therefore, new therapeutic strategies are required to improve PTC outcomes.

**Methods:**

In this study, we performed differential expression analysis using data from patients with PTC collected from the Cancer Genome Atlas program database, and prognostic analysis of differential genes. To understand the effects of ubiquitin-conjugating enzyme 9 (UBC9) on drug therapy, immunotherapy, immune relevance, and gene mutations in tumor cells of patients with PTC, we performed cancer drug susceptibility genomics, computed tumor immune dysfunction and exclusion, tertiary lymphoid tissues, cytolytic activity, immune infiltration, immune modulators, genomic signature differences, and gene ontology and Kyoto encyclopedia of genes and genomes enrichment analysis. Moreover, we investigated the function of UBC9 in tumor cells using a knockdown assay.

**Results:**

UBC9 expression level was significantly elevated in the tumor tissues of patients with PTC, and in vitro experiments demonstrated that UBC9 knockdown inhibited tumor proliferation and migration and promoted apoptosis. UBC9 is closely linked to immunity in PTC, and UBC9 may be a potential therapeutic target.

**Conclusions:**

Our study demonstrated that UBC9 is a novel therapeutic target for PTC and may be a potential strategy for its treatment.

**Supplementary Information:**

The online version contains supplementary material available at 10.1007/s40618-024-02523-y.

## Introduction

Thyroid cancer represents one of the most rapidly increasing malignancies globally, with its incidence rising by approximately 3% annually over the past three decades. According to the latest global cancer statistics (GLOBOCAN 2022), thyroid cancer accounts for 3.2% of all cancer cases worldwide, with an estimated 586,000 new cases and 43,646 deaths in 2022 [1]. Papillary thyroid cancer (PTC) is the predominant histological subtype, accounting for approximately 84–90% of all thyroid cancers worldwide [2]. This increase in incidence is particularly pronounced in younger populations, with the incidence in adolescents and young adults rising by > 4% per year [3]. Although the prognosis of most patients is good, there are still some deaths [4], mental distress, and decreased quality of life for patients [5]. Inhibition of PTC metastasis, discovery of new anticancer molecular targets, and identification of new diagnostic and therapeutic targets are key strategies to improve the prognosis of patients with PTC and develop new therapeutic agents. It is crucial to support PTC research to reveal prognostic biomarkers and advance precision therapy for PTC.

Small ubiquitin-like modifier (SUMO) modification is an essential post-translational modification that covalently attaches to SUMO homeodomain lysine residues of target proteins and is involved in the regulation of various cellular processes, including tumors, inflammation, immunity, cell cycle, and gene expression [6]. SUMOylation modification is a multistage enzymatic process in which the main enzymes involved are SUMO-activating enzyme E1, SUMO-specific coupling enzyme E2, and SUMO ligase E3 [7]. Ubiquitin-conjugating enzyme 9 (UBC9) is the most critical SUMO E2 conjugating enzyme, also known as ubiquitin-conjugating enzyme E2 I (UBE2I), which regulates numerous tumors. For example, in glioma stem cells, it was found that ubiquitin specific peptidase 34 (USP34) regulated glioma stem cell progression by directly heterodimerizing UBE2I and regulating the aberrant expression of Pin1 at the post-translational level [8]. Studies on prostate cancer have also identified an essential role for UBE2I, and ablation of UBE2I can reverse the immunosuppressive phenotype of tumor-associated macrophages, which is vital for tumor immunity [9]. Although UBE2I plays a role in different cancers, its role and mechanism of action in PTC remain unclear. Therefore, an in-depth understanding of the role of UBE2I in PTC and immune-related prognostic markers is needed to identify new targets and therapeutic strategies for treating PTC.

In this study, we focused on the correlation between UBE2I and PTC as well as the function and mechanism of UBE2I in relation to immunotherapy resistance and immunization of PTC. The differences in UBE2I expression in PTC, its prognostic significance, and its clinical value were analyzed using PTC-related data from the Cancer Genome Atlas program (TCGA) database. UBE2I was then analyzed for drug sensitivity and immunotherapeutic benefits in PTC treatment and its correlation with immune infiltration. Moreover, we screened the prognostically significant genes of patients to construct and validate prognostic models. We identified the vital role of UBE2I in PTC immunotherapy and drug sensitivity by analyzing public databases. We built a predictive model to provide substantial theoretical support for developing more effective immunotherapies.

## Materials and methods

### Data search and information

Gene expression quantification data were downloaded from RNA-seq of TCGA-THCA in the TCGA database [10], which included 59 control and 513 disease samples. All data were organized into gene expression profile form; only the genes were kept with type protein_coding, the data were log2(tpm + 1), and the same genes were taken as the mean value. Data were used as the analysis set. Samples with an overall survival time of 0 were removed, and a total of 504 disease samples with no patient duplicates were included for survival analysis.

## Differential expression and prognostic significance of UBE2I in PTC and its clinical value

Differential analysis of UBE2I expression was performed for tumor versus normal tissues based on gene expression of the analysis set data, and the significance of the difference was evaluated using a t-test. To correlate UBE2I with cancer prognosis, the R package survminer (version 0.4.9, https://cran.r-project.org/web/packages/survminer/index.html) was used to determine the optimal cutoff value, and 504 disease samples with prognostic information were categorized into high- and low-UBE2I-expressing groups. The Kaplan–Meier (KM) curve method was used to assess the correlation between the high UBE2I-expressing group (high) and the low UBE2I-expressing group (low) and the actual survival prognostic information. Only 504 samples were used for the subsequent analysis.

## Differences in UBE2I between different clinical information

The Kruskal–Wallis test [11] was used to analyze whether there was a difference in UBE2I expression among gender, M_stage, N_stage, T_stage, and stage. A correlation scatter plot was plotted to determine whether the UBE2I was correlated with age.

## Prognostic risk factor screening and nomogram construction

The UBE2I high and low subgroup information and other clinical information were combined with the sample survival prognosis information to screen for risk factors with a significant overall survival prognosis using one-way Cox regression analysis in the R package survival (version 3.5.7, http://bioconductor.org/packages/survivalr/) [12] *p*-values < 0.05 were selected as prognostic risk factors. A risk model was then constructed for the prognostic risk factors using multifactor Cox regression, and a hypothesis test was performed to observe whether the risk model was significant and could pass the hypothesis test.

A nomogram was constructed based on prognostic risk factors using the R package rms (version 6.8-0, https://cran.r-project.org/web/packages/rms/index.html) [13]. Calibration curves were plotted to estimate the discrimination and accuracy of the nomogram model, and the calibration curves were used to assess the predictive ability of the nomogram. A calibration curve was then used to assess the predictive power of the nomogram. The calibration curve demonstrated the agreement between the predicted probability of the model and the actual probability of occurrence, which was plotted using the R package rms calibration method.

## Predicting drug sensitivity and immunotherapy benefit in patients with different UBE2I subgroups

The sensitivity of each patient to chemotherapeutic agents was estimated using the Genomics of Drug Sensitivity in Cancer (GDSC https://www.cancerrxgene.org/) database [14]. The half-maximal inhibitory concentration (IC_50_) was calculated using the R package pRRophetic (version 0.5, https://github.com/paulgeeleher/pRRophetic) [15]. Differences in the IC_50_ of common chemotherapeutic agents among different UBE2I subgroups were comparatively analyzed using the Wilcoxon test.

Tumor immune dysfunction and exclusion (TIDE) was calculated using TIDE (http://tide.dfci.harvard.edu/) [16] to infer predictive potential in terms of the number of immunotherapy responses. The Wilcoxon rank sum test was used to determine whether TIDE significantly differed in high and low UBE2I subgroups.

Tertiary lymphoid tissues (TLSs) are ectopic lymphoid tissues that form at sites of prolonged inflammation, including tumors. TLSs are associated with a better prognosis in most solid tumors and can predict ICB treatment response. We used the ssGSEA algorithm of the R package GSVA (version 1.50.5, https://www.bioconductor.org/packages/release/bioc/html/GSVA.html) [17] to calculate the TLS score and the correlation with UBE2I expression.

Immune cytolytic activity (CYT) was assessed by calculating the mean values of GZMA and PRF1 expression, measurements of inflammatory indexes were performed, and correlations with UBE2I expression were calculated. Expression data of immune checkpoint genes and human leukocyte antigen (HLA) family genes were extracted. The correlation of immune checkpoint genes and HLA family genes with UBE2I was calculated using Pearson correlation.

### High and low UBE2I groups with immune infiltration

To further explore the relationship between high and low UBE2I groups and the tumor microenvironment (TME), ESTIMATE, ssGSEA, CIBERSORT, and EPIC algorithms were performed by executing ESTIMATE in R. ESTIMATE was performed using the R package ESTIMATE (version 1.0.13 https://rdrr.io/rforge/estimate/) to determine TME scores. ssGSEA scores for immune cells were calculated using the ssGSEA algorithm in the R package GSVA. The CIBERSORT and EPIC scores of the immune cells were calculated using the R package IOBR (version 0.99.8, https://github.com/IOBR/IOBR). Differences in TME and immune cell scores between high and low UBE2I groups were assessed using the Wilcoxon rank sum test.

Immunomodulator genes were obtained from TISIDB (http://cis.hku.hk/TISIDB/) [18] and analyzed for Chemokine, Immunoinhibitor, Immunostimulator, major histocompatibility complex (MHC), and Receptor, and most immunomodulators differed between high and low UBE2I expression groups.

The GSVA algorithm of the R package GSVA was used to calculate the scores for several biological processes constructed by Mariathasan et al. [19]. including immune checkpoints, antigen processing processes, CD8 + T-effector features, epithelial-mesenchymal transition (EMT) markers including EMT1, EMT2, and EMT3, angiogenesis features, pan-fibroblast TGF-β response signature, WNT targets, DNA damage repair, mismatch repair, nucleotide excision repair, DNA replication, and antigen processing and presentation. The correlation of these pathway scores with UBE2I was calculated using Pearson’s correlation, and t-tests were used to evaluate the differences in scores between the high and low UBE2I groups.

## Differences in genomic characterization between high and low UBE2I groups

Masked copy number segment data were obtained using the R package TCGA biolinks (version 2.30.4, https://bioconductor.org/packages/release/bioc/html/TCGAbiolinks.html) [20] to compare the change in recurrent copy number between high and low UBE2I expression groups using GISTIC2.0 (https://www.genepattern.org/modules/docs/GISTIC_2.0) [21] to compare the change in recurrent copy number between high and low UBE2I expression groups.

Masked somatic mutation data were downloaded from the GDC (https://portal.gdc.cancer.gov/) and the R package maftools (version 2.18.0, https://bioconductor.org/packages/release/bioc/vignettes/maftools/inst/doc/maftools.html) was used to plot a waterfall plot of mutation information.

Tumor Mutational Burden (TMB) and microsatellite instability (MSI) data were obtained using the R package TCGAplot (version 7.0.1, https://github.com/tjhwangxiong/TCGAplot), and t-tests were used to evaluate the differences in TMB and MSI between high and low UBE2I expression groups.

## UBE2I functional analysis

Differential analyses were performed on the high and low UBE2I sets using the R package limma (version 3.58.1, https://www.bioconductor.org/packages/release/bioc/html/limma.html) [22] and on the high and low UBE2I sets using the R package clusterProfiler (version 4.12.0, https://bioconductor.org/packages/release/bioc/html/clusterProfiler.html) [23] to perform GSEA enrichment analysis of differential analysis results in the hallmark and Kyoto encyclopedia of genes and genomes (KEGG) gene sets.

Genes with|log2FC|>1 and p-values < 0.05 were selected as differential genes, and gene ontology (GO) [24] and KEGG [25] functional analyses were performed on the differential genes to explore the related potential biological processes and pathways. The Benjamini–Hochberg method was used for multiple testing corrections to obtain the corrected *p*-value, i.e., adjust.pvalue. adjust.pvalue < 0.05 and count ≥ 2 was used as the threshold to screen for meaningful enrichment results.

### Screening for prognostically significant genes and constructing prognostic models

Based on the differential genes obtained from the above screening, one-way Cox regression analysis in the R package survival was utilized to screen for risk genes with significant survival prognosis, and those with a *p*-value ˂ 0.05 were selected as prognostic risk genes.

The samples were randomly divided into training and validation sets in a 6:4 ratio, stratified by survival times of 1, 3, and 5 years. In the training set, the R package glmnet (version 4.1–8, https://cran.r-project.org/web/packages/glmnet/index.html) [26] was used to screen the above risk genes according to Least absolute shrinkage and selection operator(LASSO) regression analysis to construct a new prognostic model. The risk score formula was established using the regression coefficients of each gene and the expression levels of the modeled genes as follows:

Risk score = β1 × 1 + β2 × 2 +… + βnXn.

#### Note

In this equation, β refers to the regression coefficient, and X is the value of the feature; in this case, the expression of the gene.

The risk score for each sample was calculated using this model, and the samples were categorized into high- and low-risk groups using the R package survminer to determine the optimal cutoff value. The correlation between the grouping of high-risk (HIGH) and low-risk (LOW) groups and actual survival prognostic information was assessed using the KM curve method. The predictive performance is represented by the risk maps.

### Prognostic model validation

The prognostic risk model was further validated using the validation and full sets to assess model robustness. The validation set and full set samples were categorized into high-risk group (HIGH) and low-risk group (LOW) using the R package survminer to determine the optimal cutoff value. The prediction performance is represented by a risk map.

### Cell culture and transfection

The human PTC cells BCPAP were bought from Pricella (CL-0575, China). BCPAP cells were cultured in RPMI 1640 medium containing 10% fetal bovine serum and 1% penicillin-streptomycin at 37 ℃ and 5% CO_2_ humidified incubator.

The knockdown lentiviral vector shUBE2I and the corresponding negative control were procured from GenCefe (China), and the lentiviral transfection was performed following the manufacturer’s instructions. Briefly, 5 × 10^4^ cells were grown in 6-well plates overnight and transduced with lentiviral vectors according to the manufacturer’s instructions. The cells were screened for puromycin two days after transduction for subsequent experiments.

### Western blotting

Total protein was extracted from cells and tissues using RIPA lysate (Solarbio R0020) containing 1% protease inhibitor (PMSF). Protein samples (40 µg) were separated by 10% sodium dodecyl sulfate polyacrylamide gel electrophoresis. Proteins were transferred to polyvinylidene fluoride membranes with rabbit anti-UBE2I (Abcam ab75854), murine anti-GAPDH (Proteintech 60004-1-Ig), and β-actin (Proteintech) primary antibodies overnight at 4 ℃. The secondary antibodies were prepared using horseradish peroxidase-labeled goat anti-rabbit IgG (affinity S0001), and goat anti-mouse IgG (affinity S0002) were incubated with the membrane for 2 h at room temperature. Finally, the ECL chemiluminescent solution was dropped onto the membrane for detection, and the results were analyzed using ImageJ software.

### Cell counting kit-8 (CCK-8) assay

The CCK8 kit was used to detect cell viability (Solarbio CA1210), and cells were grown in 96-well plates at 1 × 10^4^ cells/well and detected at 24, 48, and 72 h, respectively. About 10 µL of CCK8 reagent was added to each well and incubated at 37 ℃ for 2 h. The absorbance at 450 nm was detected using an enzyme marker (Thermo Fisher).

### Cell migration and invasion assays

Cell migration and invasion were detected using a 24-well 8 μm pore size transwell. Migration experiments were performed with a 200 µL density of 5 × 10^4^ transfected cells per well inoculated in the upper chamber of the transwell. After 24 h of incubation, cells that migrated under the membrane were stained with crystal violet. For invasion experiments, a matrix gel-coated transwell was used, and a 200 µL density of 5 × 10^4^ cells per well was inoculated into the upper chamber of the chambers. After 24 h of incubation, cells migrating below the membrane were stained with crystal violet. The number of stained cells was counted with an inverted microscope (Leica Z5458), five fields of view were randomly selected for counting, and the experiment was repeated thrice.

### Wound healing assay

Using transfected PTC planted in 6-well plates, a straight line was drawn with a 200 µL lance tip when the cells were completely grown, then the cells drawn down were washed with PBS, and serum-free 1640 medium was added. The photographs were taken and recorded under an inverted microscope (Leica Z5458) for 0 h and put into the incubator to continue incubation, followed by photographs retaken for the next 24 h, and the distance of the healed wounds was recorded.

### Colony formation assay

Transfected PTCs were seeded in 6-well plates at 500/well. Colony formation was seen after two weeks of incubation, fixed with 4% paraformaldehyde for 30 min, stained with 0.1% crystal violet for 30 min, and photographed and documented with honor phone. The results were analyzed using the ImageJ software to calculate the colony formation rate.

### Cell cycle and apoptosis analysis

Cells were processed and stained following the manufacturer’s instructions using a cell cycle kit (Solarbio CA1510) and an apoptosis kit (Solarbio CA1040) and subsequently assayed on a flow cytometer. Finally, cell cycle and apoptosis data were statistically analyzed using Modfit LT (version 4.0) and FlowJo software (version 10).

### Statistical analysis

All statistical analyses were performed using the GraphPad Prism software (version 9). Data were tested for compliance with the chi-square test and normal distributions. Measurements are expressed as the mean ± standard deviation. Comparisons between two groups were performed using paired or unpaired t-tests, and comparisons between multiple groups were performed using a one-way analysis of variance. Statistical significance was set at *p* < 0.05.

## Results

### UBE2I expression differences in PTC, prognostic significance, and its clinical value

**Fig. 1:**
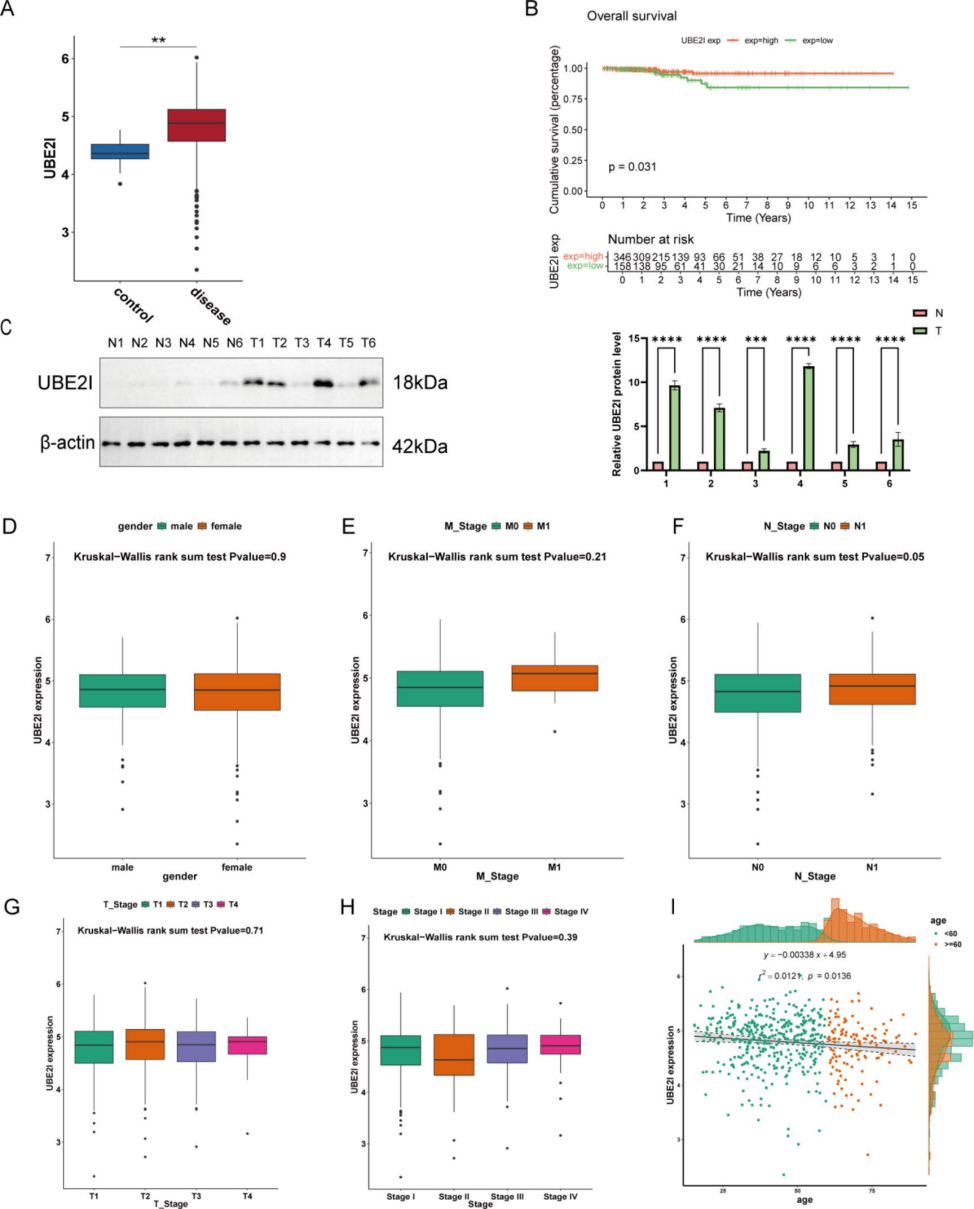
Differential expression of UBE2I in PTC cells. (A) TCGA database of UBE2I expression in normal and tumor samples. (B) KM survival curves of the high and low UBE2I subgroups. (C-D) IHC and WB analyses of UBE2I expression in tumor samples. (E–I) Changes in UBE2I expression at different PTC stages. (J) Correlation analysis of UBE2I with age of patients with PTC. *p < 0.05, **p < 0.01, ***p < 0.001, ns, non-significance

Analysis of PTC data from the TCGA database revealed that UBE2I expression was significantly elevated in tumor tissues (Fig. 1A). Using the optimal cutoff threshold of 4.63 to divide the tumor samples into the UBE2I high- and low-expression groups. KM analysis revealed that the high level of UBE2I expression was significantly correlated with the prognosis of the patients, with low expression of UBE2I contributing to the prognosis of the patients and the patients in the UBE2I high expression group having a poor prognosis (Fig. 1B). These data suggest the significance of UBE2I in the clinical diagnosis of PTC. To further validate the UBE2I expression in PTC, Western blotting (WB) validation using clinical samples revealed that UBE2I expression was significantly higher in cancer tissues than in paracancerous tissues (Fig. 1C), and the results were consistent with the data from TCGA database. Moreover, UBE2I expression was non-significantly different in different gender, M_stage, N_stage, T_stage, and stage (Fig. 1D–H). However, UBE2I expression significantly correlated with age (Fig. 1I). These results suggest that UBE2I expression is associated with PTC occurrence.

### Screening of prognostic risk factors and construction of nomogram

**Fig. 2:**
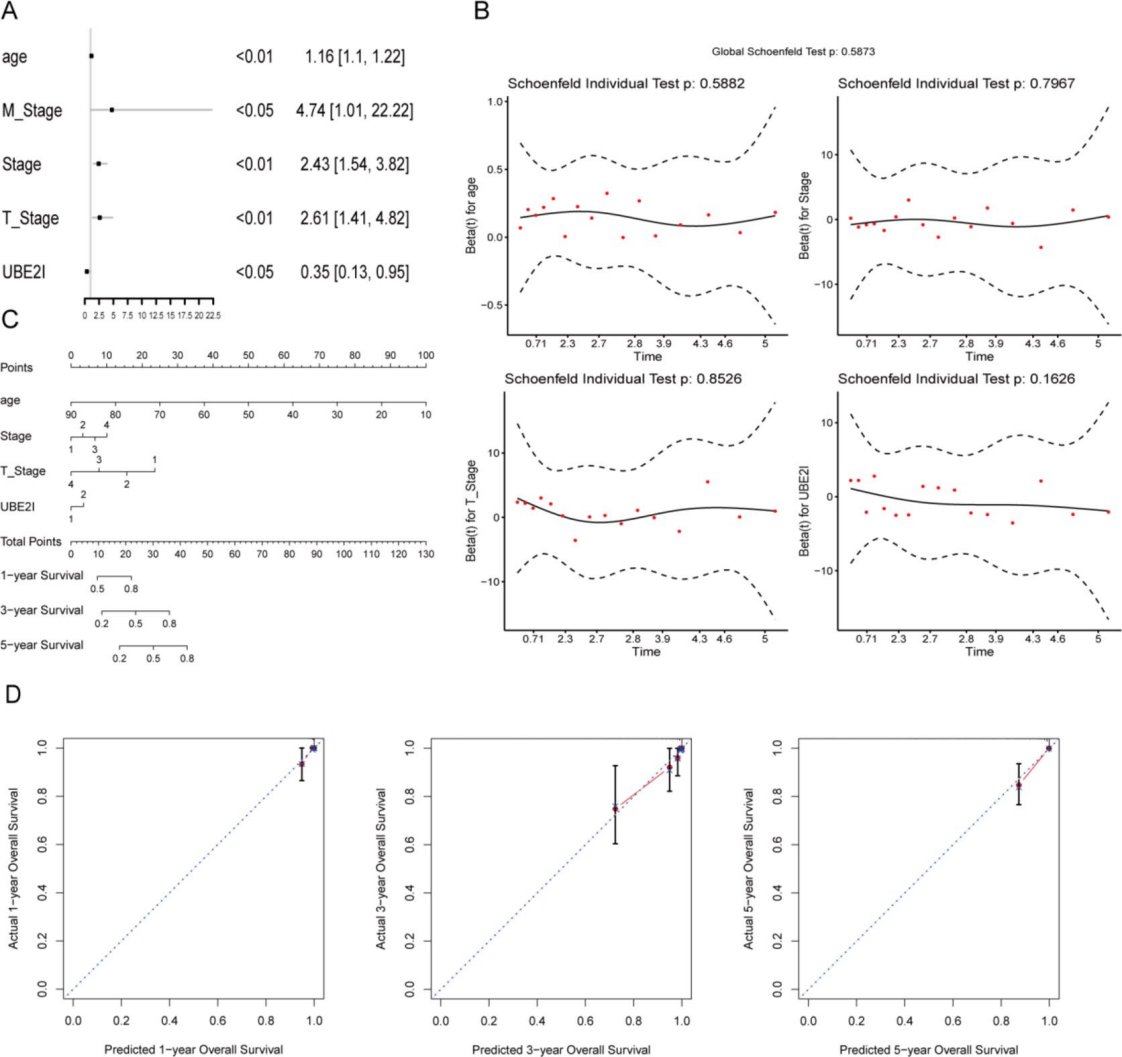
Prognostic risk factor screening. (A) Forest plot of one-way Cox regression results. (B) Schoenfeld's residuals test results. (C) Survival analysis column line plot. (D) Survival analysis nomogram calibration curve

One-way Cox regression analysis revealed that age, M_stage, stage, T_stage, and UBE2I were significantly associated with patient survival (Fig. 2A). Due to the excessive missing values of M_Stage, M_Stage was excluded, and the remaining four prognostic risk factors were analyzed by multifactorial Cox regression analysis followed by Schoenfeld’s residuals test (Fig. 2B). All tests resulted in a *p*-value > 0.05, indicating that the effect of the covariates on survival did not change over time. Nomograms were constructed using AGE, stage, T_stage, and UBE2I (Fig. 2C), and the calibration curves of the 1-, 3-, and 5-year column line plots all overlapped the diagonal line to a high degree, indicating good agreement (Fig. 2D).

#### Drug sensitivity and immunotherapeutic benefit in patients in high and low UBE2I subgroups

**Fig. 3:**
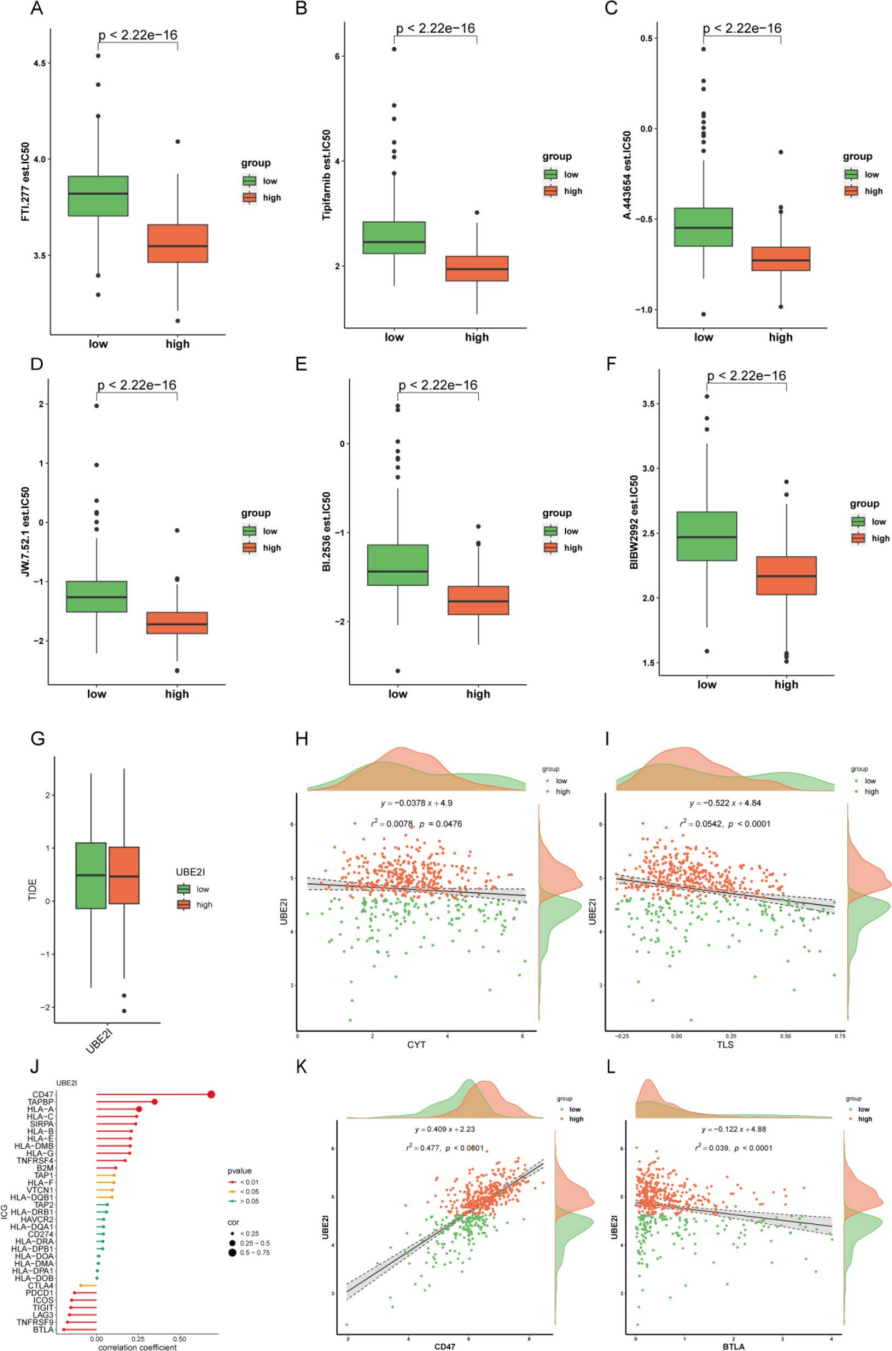
A–F) IC50 levels of the top six chemotherapeutic agents with the most significant differences. (G) UBE2I high and low subgroups correlate with TIDE. (H) TLS score and UBE2I correlation scatter plot. (I) CYT score and UBE2I correlation scatter plot. (J) Immune checkpoint genes and HLA family 391 genes correlating with UBE2I lollipop plot. (K) CD47-UBE2I correlation scatter plot. (L) BTLA-UBE2I correlation scatter plot

The sensitivity of each patient to chemotherapeutic drugs was estimated based on the GDSC database, and IC_50_ was quantified using the R package pRRophetic. Patients with high UBE2I had lower IC_50_ values for FTI.277, tipifarnib, A.443,654, JW.7.52.1, BI.2536, and BIBW2992 (Fig. 3A–F). These drugs target key oncogenic pathways including the RAS/MAPK signaling (FTI.277 and tipifarnib), PLK1 (BI.2536), and EGFR (BIBW2992) pathways, suggesting that UBE2I may serve as a predictive biomarker for response to these targeted agents. A comparison of TIDE scores revealed non-significant differences between the high and low subgroups of UBE2I (Fig. 3G). We further analyzed the correlation between tertiary lymphoid structure (TLS) and CYT scores and UBE2I expression and found that UBE2I was significantly correlated with TLS and CYT (Fig. 3H-I).Moreover, given that intracellular pathway modulation has been successfully employed in prostate cancer treatment [27], development of specific UBE2I inhibitors could provide a novel therapeutic approach. The therapeutic potential of targeting UBE2I is further supported by our observation that UBE2I modulates multiple oncogenic signaling pathways including NF-κB signaling and inflammatory responses. This multi-targeted approach, similar to successful strategies in prostate cancer [28], could help overcome treatment resistance.

Correlation analysis of the immune checkpoint genes and HLA family genes with UBE2I revealed that 22 immune checkpoint genes and HLA family genes were significantly correlated with UBE2I (Fig. 3J). The highest positive correlation was observed for CD47-UBE2I, with a correlation coefficient of 0.69, a squared correlation coefficient of 0.477, and a correlation *p*-value < 0.0001 (Fig. 3K). The highest negative correlation was observed for BTLA-UBE2I, with a correlation coefficient of − 0.20, a squared correlation coefficient of 0.039, and a correlation *p*-value < 0.0001 (Fig. 3L). The above results suggest a strong correlation between UBE2I and PTC immunization.

### Correlation of high and low UBE2I groupings with immune infiltration

**Fig. 4:**
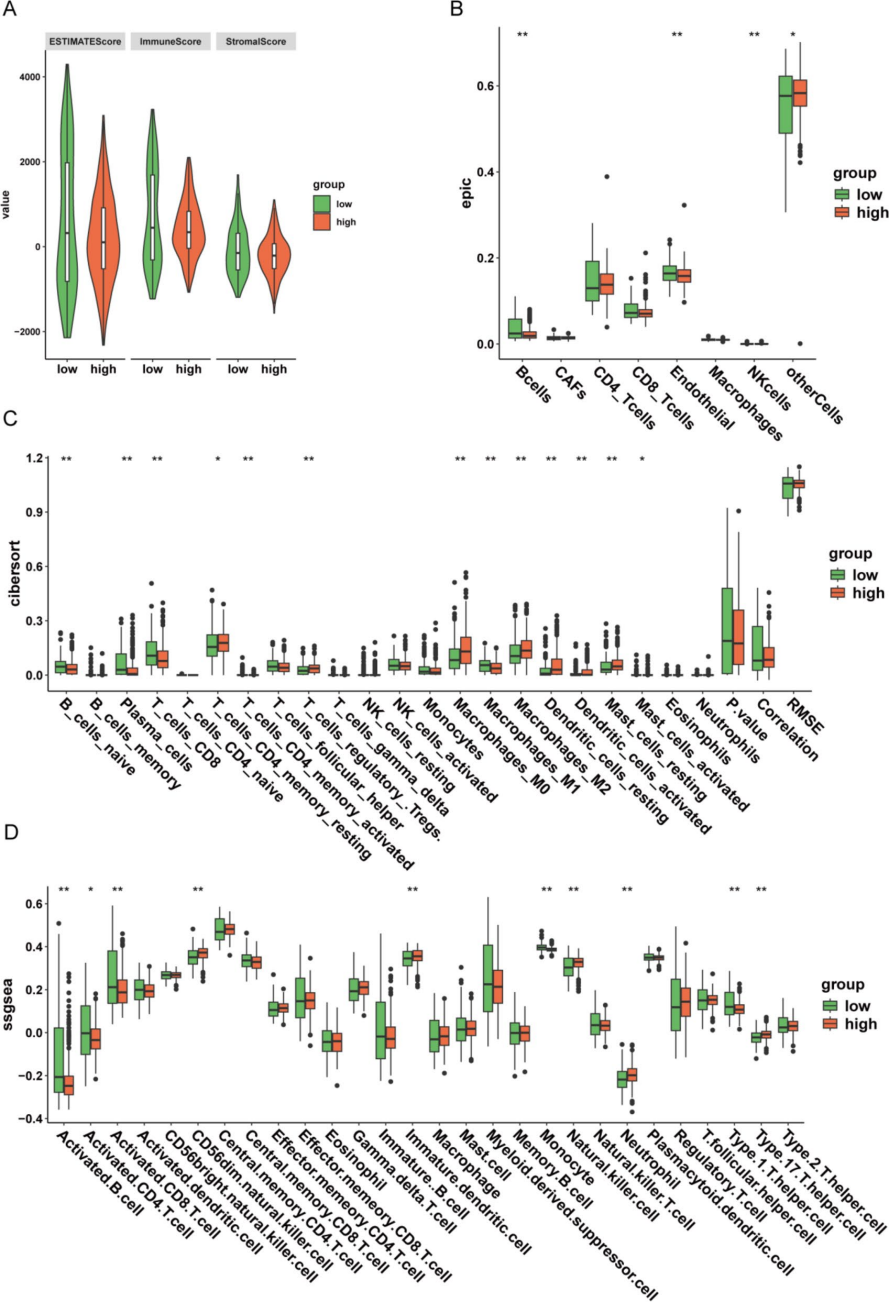
Analysis of UBE2I correlation with immune cells. (A) Violin plot depicting TME score. (B) Immune cell EPIC score box line plot. (C) Immune cell CIBERSORT score box line plot. (D) Immune cell SGSEA score box line plot. *p < 0.05, **p < 0.01, ***p < 0.001, ns, no significance

We used ESTIMATE to assess TME scores and found non-significant differences in any of the three metrics between the high and low UBE2I groups (Fig. 4A). Our scoring of immune cells using EPIC, CIBERSORT, and ssGSEA revealed significant differences in immune cells, such as B cells, endothelial cells, NK cells, plasma, CD4 T cells, CD8 T cells, and macrophages between the high and low UBE2I expression groups (Fig. 4B–D).

**Fig. 5:**
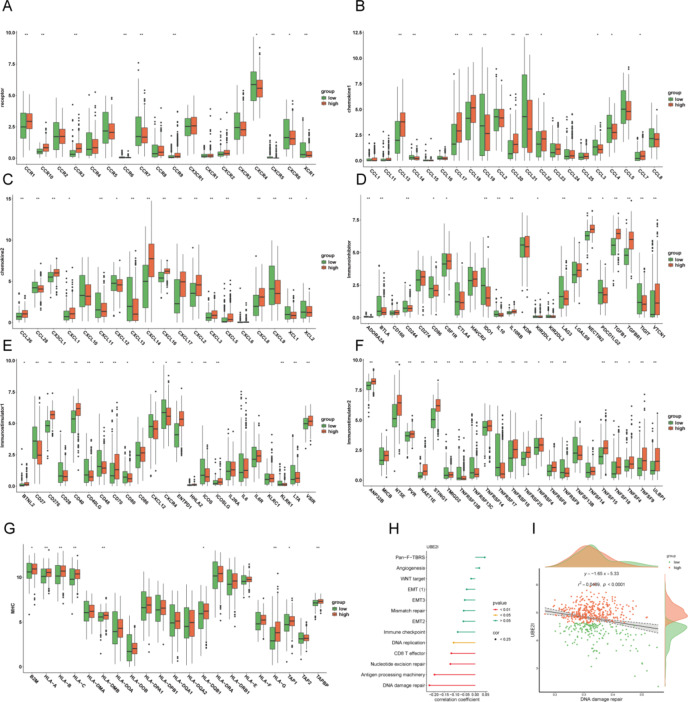
UBE2I correlation analysis with immunomodulators. (A) Receptors correlating with UBE2I. (B-C) Chemokines correlating with UBE2I. (D) Immunosuppressants correlating with UBE2I. (E-F) Immunoenhancers correlating with UBE2I. (G) Major histocompatibility complexes correlating with UBE2I. (H) Correlation lollipops. (I) DNA damage repair- UBE2I correlation400 scatter plot

Moreover, we analyzed the difference between the high and low UBE2I expression groups for most immunomodulators, including chemokines, immunoinhibitors, immunostimulators, MHC, and receptors. We found that most immunomodulators were significantly different between the high and low UBE2I expression groups. All modulators depicted significant differences between the high and low UBE2I expression groups (Fig. 5A–E).

We subjected several biological processes constructed by Mariathasan et al. to GSVA scoring and calculated the correlations with UBE2I. We found that no processes were significantly positively correlated with UBE2I, and five processes were significantly negatively correlated with UBE2I. The strongest negative correlation was DNA damage repair-UBE2I, with a correlation coefficient of − 0.22 and a squared correlation coefficient of 0.05 (Fig. 5H).

#### Differences in genomic characterization between high and low UBE2I groups

**Fig. 6:**
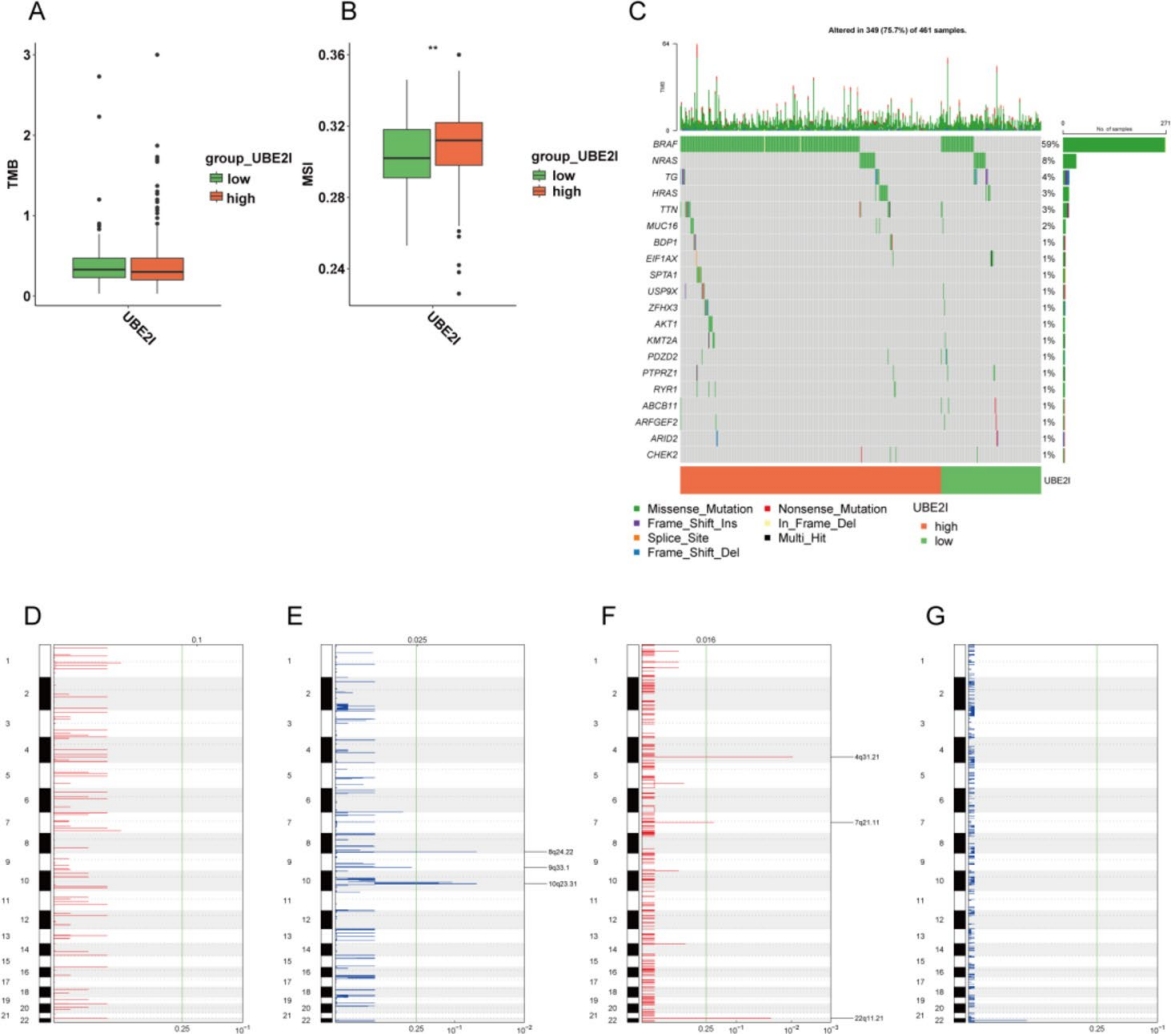
UBE2I and genomic characterization. (A) TMB correlates with UBE2I. B BMSI versus UBE2I correlation. C Mutation waterfall plot. (D–E) GISTIC results of copy number variation in the UBE2I low-expression group. (F-G) GISTIC results of copy number variation in the UBE2I high-expression group. *p < 0.05, **p < 0.01, ***p < 0.001, ns, no significance

TMB and MSI analyses of the high and low UBE2I subgroups revealed non-significant differences in TMB between the high and low UBE2I subgroups and a significant difference in MSI between the high and low UBE2I subgroups (Fig. 6A). The mutation waterfall map is displayed in Fig. 6C, and the GISTIC in the UBE2I low expression group had significant deletions in the regions of 8q24.22, 9q33.1, and 10q23.31 (Fig. 6D). The GISTIC in the UBE2I high expression group had significant amplifications in 4q31.21, 7q21.11, and 22q11.21 (Fig. 6F). Our findings revealed a correlation between UBE2I and PTC gene mutations, suggesting a potential impact of these mutations on tumor progression and immunotherapy.

#### UBE2I functional enrichment analysis

**Fig. 7:**
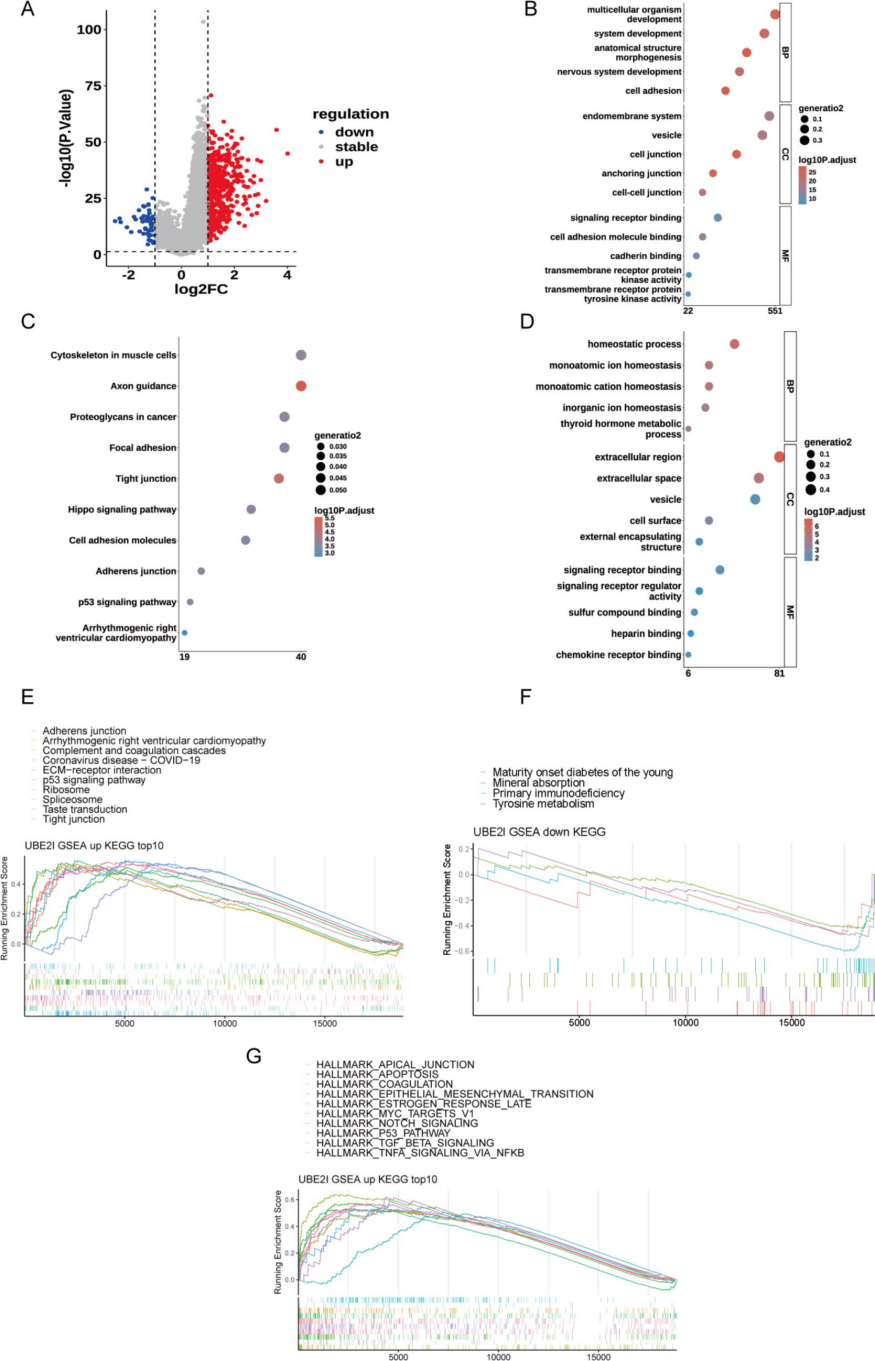
UBE21-related gene enrichment analysis. (A) High and low UBE2I group difference analyses. (B) Upregulated differential gene GO enrichment analysis. (C) Upregulated differential gene KEGG enrichment analysis. (D) Downregulated differential gene GO enrichment analysis. (E) Top 10 most significantly upregulated KEGG pathway GSEA enrichment maps. (F) Downregulation of the top four most significant KEGG pathway GSEA enrichment maps. (G) Upregulation of the top 10 most significant hallmark pathway GSEA enrichment maps

Further differential analysis of the high and low UBE2I groups yielded 455 differentially upregulated genes and 54 differentially downregulated genes (Fig. 7A). These upregulated and downregulated genes were then analyzed by GO and KEGG enrichment analyses, respectively.

With p.adjust < 0.05 and count > = 2, there were 1122 significantly enriched GO items for upregulated differential genes, including 875 BP classes (the most significant item was anatomical structure morphogenesis), 166 CC classes (the most significant item was anchoring junction), 166 CC classes (the most significant item was anchoring), and 81 MF classes (the most significant item was cell adhesion molecule binding). There were 24 significantly enriched KEGG items (the most significant item was the axon guidance). The bar graphs depicted the top five GO enrichment analysis results with the most significant p.adjust in each category of GO enrichment analysis (Fig. 7B) and the top 10 results with the most significant p.adjust for KEGG enrichment analysis (Fig. 7C).

With p.adjust < 0.05 and count > = 2, there were 199 significantly enriched GO items for downregulated differential genes, including 166 BP classes (the most significant item was homeostatic process), 7 CC classes (the most significant item was extracellular region), 26 MF classes (the most significant item was signaling receptor binding). There were no significantly enriched KEGG items. The bar graphs exhibited the top five most significantly enriched GO enrichment analysis results for p.adjust in each category of GO enrichment analysis (Fig. 7D).

The differential analysis results were analyzed using hallmarks and GSEA of KEGG gene sets. Twenty-six significant KEGG pathways were obtained, including 22 upregulated pathways (Fig. 7E) (the most significant being tight junction) and 4 downregulated pathways (the most significant being primary immunodeficiency) (Fig. 7F).

Ten significant hallmark pathways were obtained, including 20 upregulated pathways (most notably HALLMARK_COAGULATION) and 0 downregulated pathways (Fig. 7G).

#### Screening patients for prognostically significant genes and constructing prognostic models

**Fig. 8:**
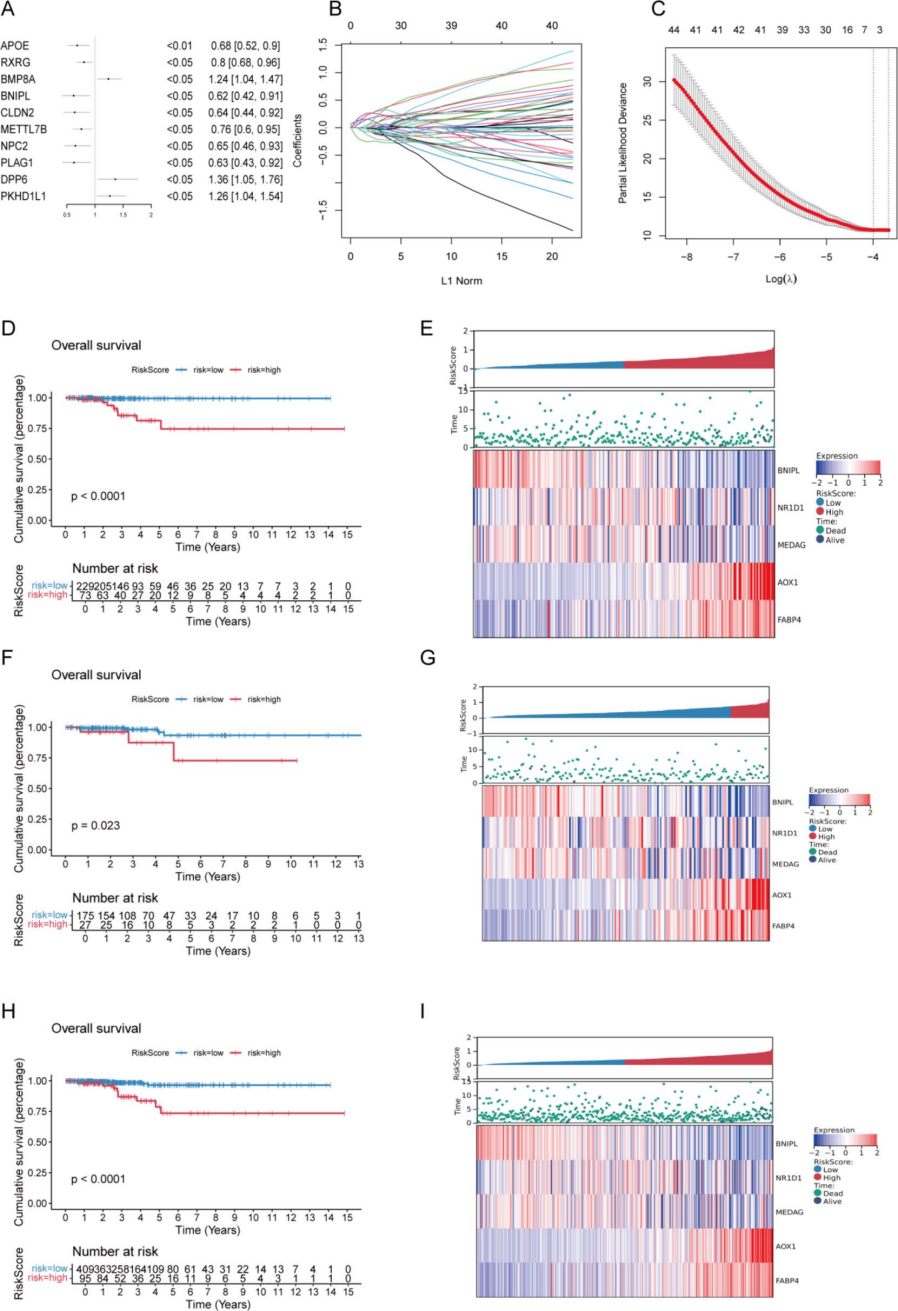
Prognostic gene screening and prognostic model construction. (A) Top 10 most significant prognostic genes. (B) LASSO coefficient distribution plot. (C) Likelihood deviation of the LASSO coefficient distribution. (D) Training set survival curve. (E) Training set risk plot. (F) Validation set survival curve. (G) Validation set risk plot. (H) Full set survival curve. (I) Full set risk plot

Screening for genes significantly associated with prognosis among the differential genes obtained above. A total of 54 prognostically significant genes were obtained using one-way Cox regression analysis. The forest plot demonstrated the top 10 most significant genes (Fig. 8A). The above prognostically significant genes were screened in the training set using LASSO. Five genes were obtained (Fig. 8B), and then the LASSO regression coefficients of these five genes were used as the prognostic model. The risk scores of each sample in the training set were calculated by combining the expression values of these five genes. The training set samples were classified into high-risk groups according to the optimal threshold of 0.62 (high) and low-risk group (low). The KM survival curve graphs of the HIGH and LOW groups exhibited that the prognosis of the high-risk group was poorer (Fig. 8D), and the model risk graph is displayed in Fig. 8E.

The model was then applied to the validation set, and the validation set samples were divided into a high-risk group (high) and a low-risk group (low) according to the optimal threshold value of 0.73. The KM survival plots of the high and low groups illustrated a significant difference in the survival status of patients in the high- and low-risk groups (Fig. 8F). The modeled risk plot is depicted in Fig. 8G.

By applying the model to the full set, the full set samples were divided into a high-risk group (high) and a low-risk group (low) according to the optimal threshold of 0.67. The KM survival curve plots of high- and low-risk groups depicted that there was a significant difference in the survival status of the patients in the high- and low-risk groups (Fig. 8H), and the model risk plot is depicted in Fig. 8I. The above results reveal that our model can categorize the samples in the training set and validation full set into high- and low-risk groups with good efficacy.

### UBE2I knockdown inhibited PTC cell proliferation and migration and promoted apoptosis

**Fig. 9:**
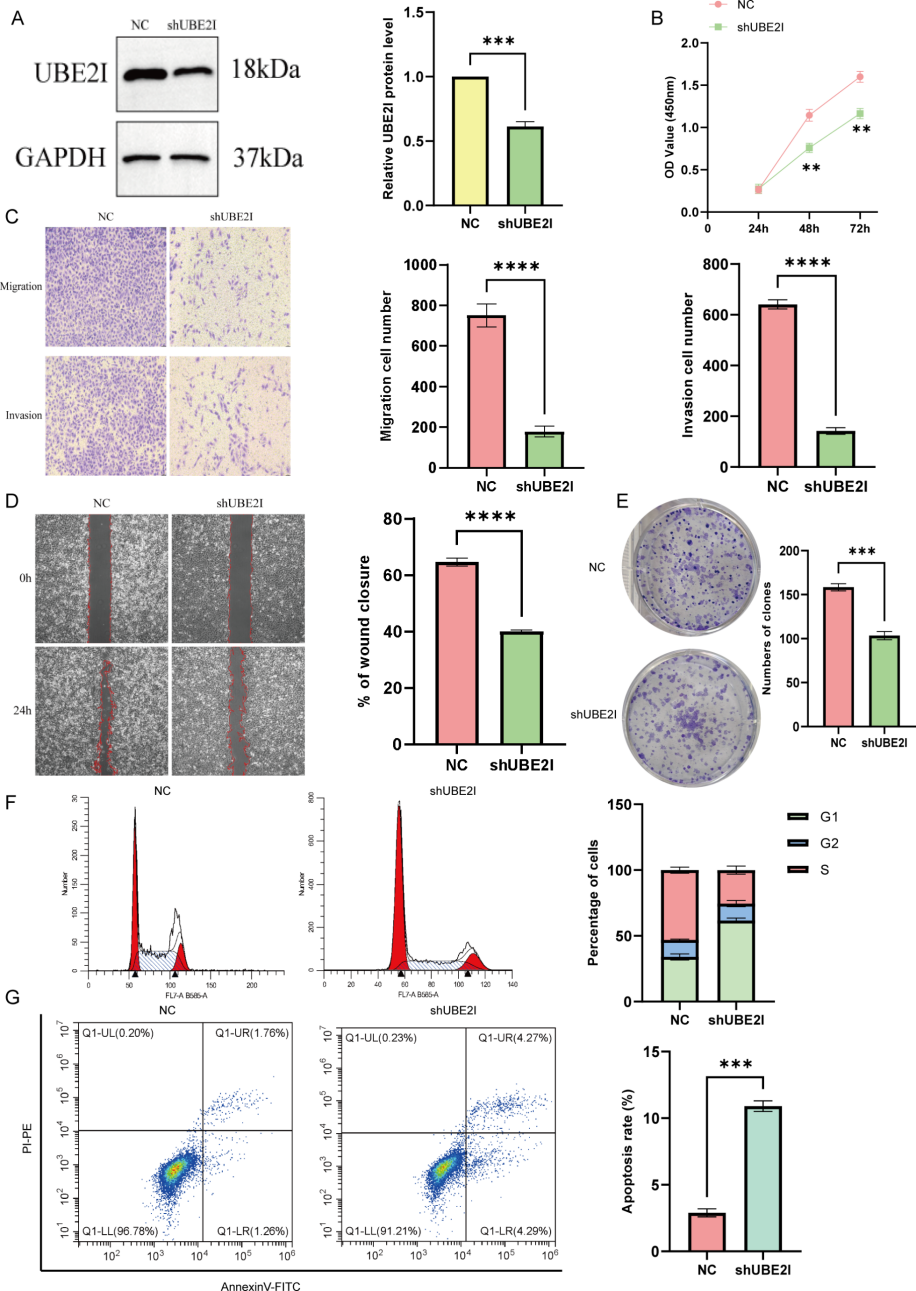
In vitro, knockdown of UBE2I inhibits proliferation, migration, and invasion of PTC cells. (A) The efficiency of NDRG1 was verified in HCC cells. (B) CCK-8 assay. (C) Transwell assay. (D) Wound healing assay. (E) Colony formation assay. (F) cell cycle assay. (G) cell apoptosis assay.

We performed UBE2I knockdown in BCPAP cells and verified the results by immunoblotting (Fig. 9A). CCK8 (Fig. 9B) and colony formation assay (Fig. 9E) revealed that UBE2I knockdown significantly inhibited the BCPAP cell proliferation. Transwell assay revealed that the migration and invasion of BCPAP cells were significantly reduced by UBE2I knockdown (Fig. 9C), and scratch assay (wound healing assay) (Fig. 9D) was consistent with the migration assay. The subsequent Transwell assay revealed that the migration and invasion ability of BCPAP cells were significantly decreased after UBE2I knockdown (Fig. 9C), and the wound healing assay (Fig. 9D) was consistent with the migration assay results. Subsequently, cell cycle and apoptosis assays were performed on UBE2I knockdown cells, and it was found that UBE2I knockdown blocked the cell cycle at the G1 phase and significantly promoted cell apoptosis. In summary, UBE2I can promote PTC cell proliferation, migration, and invasion.

## Discussion

SUMOylation pathway plays a crucial role in various cancers, including PTC. Our study demonstrates that UBE2I, as the sole E2-conjugating enzyme in SUMOylation, is significantly upregulated in PTC tissues. This finding aligns with previous studies investigating other components of the SUMOylation pathway in PTC. For instance, SUMOylation has been reported to be overexpressed in PTC tissues and correlates with tumor size and lymph node metastasis [29]. Additionally, SUMOylation plays a crucial role in regulating tumor cell proliferation and migration; studies have found that SUMO modifications may enhance tumor invasiveness by influencing signaling pathways in tumor cells [30].

The role of UBE2I varies across different cancer types, exhibiting context-dependent functions. For example, during the carcinogenesis mediated by human papillomavirus (HPV), the expression of UBE2I gradually increases, indicating its importance in cancer progression [31]. Furthermore, the absence or overexpression of UBE2I can affect the function of pancreatic β cells, leading to the onset of diabetes, which further underscores its significance in cellular functional regulation [32]. In colorectal cancer, UBE2I has been found to inhibit cancer progression by promoting the ubiquitination and degradation of RPS3. Downregulation of UBE2I is associated with poor prognosis in colorectal cancer tissues and cell lines, suggesting its potential as a tumor suppressor [33]. Additionally, the SUMOylation function of UBE2I plays a crucial role in regulating cellular resistance to oxidative stress, a mechanism that may be achieved by modulating NRF2 activity, thereby enhancing the cell’s ability to clear reactive oxygen species [32]. The function of UBE2I is not limited to the SUMOylation process within cells; it is also closely related to the regulation of multiple signaling pathways. For example, Ubc9 plays a crucial role in regulating important signaling pathways such as NF-κB and p53, which are closely associated with processes such as cell cycle, apoptosis, and transcription [34]. Therefore, abnormal expression of UBE2I may lead to dysregulation of these signaling pathways, thereby promoting the occurrence and development of tumors.

We analyzed UBE2I expression in PTC for the first time and linked it to patient prognosis, drug screening, and immunomodulation. Based on our findings and prognostic risk assessment, patients in the UBE2I high-expression group had a poor prognosis, suggesting that UBE2I may be a predictive marker for PTC risk. SUMOylation has been depicted to be associated with PTC, and predictive markers are associated with SUMO on PTC [35]. UBE2I, an essential enzyme in SUMOylation, is involved in cellular stress, cellular growth, senescence or apoptosis, cancer development [36], and processes such as autophagy [37]. Although UBE2I is closely associated with disease and cancer development, its exact mechanism and biological functions remain incompletely understood. Therefore, further studies are needed to investigate the role of UBE2I in disease and its occurrence mechanism and identify it as a potential therapeutic target. However, the role of UBE2I in PTC has not been previously reported. To determine the role of UBE2I in PTC, this study mined data from the TCGA database. It analyzed the correlation between UBE2I and PTC in the TCGA database to explore the potential mechanism of action of UBE2I in PTC.

Bioinformatics analysis revealed that UBE2I expression was significantly elevated in PTC tissues and that patients in the UBE2I high-expression group had a poorer prognosis. Validation using clinical samples also yielded the same results, suggesting that UBE2I may be important for the prognosis of patients with PTC. Our results found that downregulation of UBE2I inhibited proliferation, migration, invasion, and cell cycle of PTC cells and promoted apoptosis. To further demonstrate the importance of UBE2 in PTC, we analyzed the correlation between UBE2I and clinical information on PTC and found that UBE2I was significantly correlated with patient age. These results suggest that UBE2I expression may be associated with the occurrence of PTC. The occurrence of PTC involves complex clinical factors [38]. To predict the clinical factors and patient prognosis, we constructed a nomogram of UBE2I associated with different clinical factors to predict the survival probability of patients. Due to tumor heterogeneity remodeling of the tumor microenvironment [39], individual differences in drug sensitivity and resistance lead to drug therapy failure and greatly limit the use of drugs in oncology clinics [40]. To evaluate and screen for UBE2I-related sensitive drugs, we screened sensitive drugs in patients in the UBE2I high-expression group.

The tumor immune microenvironment is very complex, and tumors differ from other diseases in that they evolve from the host and within the body and escape immune control by the time the tumor is clinically active. Surviving tumor cells accumulate intrinsic defenses and take up other cells, including immune cells, to support escape [41]. Immunity is critical for understanding the progression of tumorigenesis and guiding immunotherapy. Therefore, we performed UBE2I-related immunotherapy and immune infiltration correlation analysis and found that UBE2I was significantly correlated with TLS and CYT. Contrarily, 22 immune checkpoint genes and HLA family genes were significantly correlated with UBE2I. Correlation analysis with the tumor microenvironment revealed that the expression of immune cells, such as B cells, endothelial cells, NK cells, plasma, CD4 T cells, CD8 T cells, and macrophages, varied significantly between the high and low UBE2I expression groups. Furthermore, the correlation between immune-related immunomodulators and UBE2I was analyzed, and it was found that most immunomodulators correlated with UBE2I expression in PTC.

Further analysis of the relationship between UBE2I and specific immune cell populations revealed significant correlations with regulatory T cells (Tregs) and CD8 + T cells. High UBE2I expression was associated with increased infiltration of Tregs, suggesting a potential immunosuppressive mechanism. Meanwhile, CD8 + T cell levels showed a negative correlation with UBE2I expression, indicating that UBE2I might contribute to T cell exclusion in the tumor microenvironment. Interestingly, the interaction between UBE2I and thyroid hormone signaling may play a crucial role in modulating the immune microenvironment. Recent studies Thyroid hormones act synergistically with androgen signalling to drive immune imbalance and cancer progression in the tumour microenvironment by regulating deiodinase activity, enhancing pro-inflammatory signalling and metabolic reprogramming [27]. Thyroid hormones can enhance the activity of T cells and promote their recruitment to sites of inflammation, which is crucial for the body’s ability to resist infections and tumors [42, 43]. Our analysis revealed a correlation between UBE2I expression and thyroid hormone metabolic process, suggesting a potential mechanism by which UBE2I might influence immune cell function through thyroid hormone-dependent pathways.

TMB is a biomarker that assesses the number of somatic mutations in the tumor genome. TMB has been used as a predictor of the response to immune checkpoint inhibitors in multiple cancer types [44]. Several studies have depicted that patients with higher TMB tend to have better outcomes when treated with therapies targeting programmed death ligand 1. MSI is a phenomenon of genomic instability that occurs mainly during DNA replication and is caused by insertion or deletion mutations during DNA replication. Detection of MSI is commonly used in the diagnosis, prognosis, and selection of therapeutic regimens for cancer, and MSI has been used in various cancer prediction and research studies [45–47]. MSI is crucial in cancer treatment and scientific research. In this study, we used bioinformatics to analyze the tumor mutation load and microsatellite instability associated with UBE2I and PTC. We found a non-significant difference in TMB between high and low subgroups of UBE2I. However, there was a significant difference in MSI between the high and low subgroups of UBE2I, suggesting a correlation between UBE2I and PTC gene mutations.

We performed GO and KEGG enrichment analyses of upregulated and downregulated genes associated with UBE2I in PTC. GO analysis of upregulated differential genes revealed that they were mainly enriched in anatomical structure morphogenesis, anchoring junction, cell adhesion molecule binding, homeostatic process, and extracellular region. KEGG analysis was mainly associated with axon guidance and tight junction signaling pathways. Downregulated differential gene GO analysis exhibited predominant enrichment in the homeostatic process, extracellular region, and signaling receptor binding, and KEGG was not enriched in the signaling pathway. Furthermore, we screened the differential genes for prognosis-related genes. We analyzed the prognostic genes to build a prognostic model, which depicted that the high-risk group had a poorer prognosis. Subsequently, we applied the model to the validation and full sets of the analysis. The results revealed significant differences in the survival status of patients in the high- and low-risk groups, and the results demonstrated the feasibility of using our model for prediction.

Although our study provides compelling evidence for UBE2I as a therapeutic target in PTC, several limitations should be acknowledged. First, while our analyses demonstrated the prognostic value of UBE2I, the cohort size is relatively modest, and validation in larger, multi-institutional cohorts would strengthen these findings. Additionally, the retrospective nature of the TCGA data analysis may introduce inherent biases.

A second limitation is that our in vitro experiments primarily focused on a single cell line. Although the results were consistent across multiple assays, validation in additional thyroid cancer cell lines and primary cultures would provide more robust evidence. Furthermore, while our knockdown experiments demonstrated the functional importance of UBE2I, overexpression studies would complement these findings and provide additional mechanistic insights.

The translational potential of our findings requires several additional steps. First, development of specific UBE2I inhibitors would be crucial for therapeutic applications. While our study suggests UBE2I as a promising target, the feasibility of selectively inhibiting UBE2I without affecting other SUMO pathway components needs careful evaluation. Second, although we observed significant correlations between UBE2I and immune responses, more detailed mechanistic studies are needed to fully understand how UBE2I modulates the tumor immune microenvironmen.

Future clinical validation studies should address several key aspects: (1) prospective evaluation of UBE2I as a biomarker in PTC patients, particularly in relation to treatment response and survival outcomes; (2) development and testing of UBE2I inhibitors, including careful assessment of safety and efficacy in preclinical models; and (3) investigation of potential combination strategies, particularly with existing standard-of-care treatments and immunotherapy.

In summary, our results exhibited that UBE2I expression was significantly correlated with PTC development in patients with PTC, with a prognostic value for clinical diagnosis. This demonstrated the key role of UBE2I in PTC development, which has been identified as a key molecular biomarker in PTC pathogenesis and may serve as a potential therapeutic target in the clinic.

## Electronic supplementary material

Below is the link to the electronic supplementary material.

Supplementary Material 1

## Data Availability

The datasets used and/or analyzed during the current study are available from the corresponding author upon reasonable request.
