# Supplementary material for: UBC9: a novel therapeutic target in papillary thyroid carcinoma
Source: J Endocrinol Invest. 2025 Mar 1;48(5):1101–19. doi: 10.1007/s40618-024-02523-y (PMC12049315; doi:10.1007/s40618-024-02523-y)
Supplement: Supplementary file 1 — Supplementary Material 1 [file 40618_2024_2523_MOESM1_ESM.docx]

Dear Editors :

Thank you for your letter and the comments concerning our manuscript. We have studied comments carefully and have made a correction which we hope meet with approval. The main corrections to this paper and responses to reviewers are as follows:

Responds to the comments:

**Reviewer #1:**

1. The criteria chosen for the differential analyses are not properly correct. In a differential gene expression analysis is necessary to adopt more stringent criteria. A safe |Log2 Fold Change| (|Log2 FC|) ≥ 1.5 or more is normally adopted as ideal cutoff, with a False Discovery Rate (FDR)/p-value < 0.05. Would the choice of |Log2 Fold Change| (|Log2 FC|) ≥ 1.0 impact the overall analysis? This question must be answered, and it would be appropriate to repeat the UBE2I functional analysis.

**Response:** Thank you very much for the reviewer’s valuable suggestion regarding the selection of thresholds in the differential gene expression analysis. We have carefully evaluated the appropriateness of using |log2 Fold Change| (|log2 FC|) ≥ 1.0 as our threshold, referencing related literature and current research practices. Below is our detailed response:

**Rationale for Selecting |log2 FC| ≥ 1.0:** We adopted the threshold of |log2 FC| ≥ 1.0, corresponding to Fold Change (FC) > 2 or FC < 0.5. This threshold is widely used in differential gene expression studies and effectively identifies genes with significant biological relevance.

Several peer-reviewed studies in the field of papillary thyroid carcinoma (PTC) and other cancers have also applied the same criteria (|log2 FC| ≥ 1.0, FDR/p-value < 0.05) for functional analysis, aligning with the standards used in our study.

**Discussion on |log2 FC| ≥ 1.5:** While we acknowledge that a higher threshold (e.g., |log2 FC| ≥ 1.5) could enhance the statistical significance of the results, it may also exclude genes with moderate expression changes that are biologically significant.

Since PTC involves diverse molecular mechanisms, genes with moderate expression changes may play a crucial role in UBE2I-associated regulatory pathways. Therefore, using |log2 FC| ≥ 1.0 provides a balanced approach, maximizing sensitivity without compromising specificity.

**Justification for the Current Methodology:** Our study aims to comprehensively explore UBE2I-related potential functions and pathways rather than focusing solely on genes with the highest expression differences. The |log2 FC| ≥ 1.0 threshold combined with FDR/p-value < 0.05 is suitable for achieving this research objective.

This choice ensures robust data quality and reliability while including more genes potentially associated with UBE2I, thereby maximizing the scientific value of the study.

**To validate the rationality of this threshold choice, our findings are supported at multiple levels:** Experimental validation of key gene UBE2I expression differences. Functional experiments confirming the biological role of UBE2I. Clinical correlation analyses supporting the clinical significance of our findings.

**References and Practical Applications:** We have supplemented our revision with references to support the appropriateness of this analysis standard and highlighted other studies that use similar criteria.(PMID: 38529286 PMID: 38154234 PMID: 38213893)

2. The authors used only one type of thyroid cancer cell line, the PCPAP cells (https://www.cellosaurus.org/CVCL_0153) as model of Poorly differentiated Thyroid gland Carcinoma (PTC). The mutational status of PCPAP cell line in BRAF (p.Val600Glu; c.1799T>A) and TP53 (p.Asp259Tyr; c.775G>T) genes could impact the responsiveness to UBE2I silencing in term of proliferation, migration and invasion. To answer and overcome this point it would be appropriate: 1. To use almost another PTC cell line wild type for the BRAF and TP53 genes, i.e. TPC1 cells (https://www.cellosaurus.org/CVCL_6298) and 2. To compare such effects in a normal human thyroid cell line, i.e. Nthy-ori 3-1 cells (https://www.cellosaurus.org/CVCL_2659).

**Response:** First, we noticed that the reviewer mentioned PCPAP cells, which we presume refers to BCPAP cells. BCPAP cells are the papillary thyroid carcinoma (PTC) cell line actually used in our study and are widely employed as a model in PTC research.

We fully recognize the potential influence of BRAF and TP53 mutations on the responsiveness to UBE2I silencing in BCPAP cells. However, we would like to emphasize the following points regarding the use of BCPAP cells in this study:

**BCPAP as a reliable PTC model:** BCPAP is a well-characterized and widely used cell line in PTC research. A substantial number of published studies have validated its reliability as a representative model for PTC. Furthermore, the BRAF V600E mutation is present in approximately 60% of clinical PTC cases, making BCPAP a clinically relevant model that reflects the genetic background of a significant proportion of PTC patients.

**Support from bioinformatics analysis:** Our study findings are further supported by comprehensive bioinformatics analyses using the TCGA database, which includes PTC samples with diverse genetic backgrounds. The consistent results between our cellular experiments and bioinformatics analyses strongly suggest that the impact of UBE2I is not limited to specific mutational profiles, such as those of BRAF or TP53.

While we acknowledge the value of using additional cell lines, such as TPC1 and Nthy-ori 3-1, to validate our findings, we believe that our current study provides robust insights into the role of UBE2I in PTC. The combination of in vitro experiments and large-scale clinical data analysis strengthens the translational relevance of our conclusions.

3. For inhibition experiments, the authors used a single short-hairpin RNA sequence for UBE2I silencing. To avoid off-target effects, it needs to use at least two different short hairpin RNA sequences and test them alone or in combination.

**Response:** We fully agree with the suggestion to use multiple shRNA sequences to validate the specificity of UBE2I silencing, which is indeed an important step to enhance the reliability of our findings.

However, I must respectfully ask for your understanding, as I have now transitioned to a new institution for my doctoral research, and it is currently not feasible for me to conduct additional experiments. While we acknowledge this as a limitation of the current study, we believe that our findings still hold significant value. They provide initial evidence for the role of UBE2I in PTC, supported by bioinformatics analyses and the functional experiments we have completed.

4. The UBE2I protein expression level in tumor tissues (Figure 1C) are not consistent with the proposed concept that UBE2I in over-expressed in tumor compared to non-tumor PTC tissues. Considering the UBE2I/β-actin ratio, about the 50% of tumor tissues showed in the Western Blot (T3-T5-T6) have UBE2I protein expression levels similar to normal PTC tissues. The authors should consider extending the analysis using other tumoral samples.

**Response:** We would like to thank the reviewer for their valuable comments. We would like to clarify the concerns raised regarding the UBE2I protein expression and provide a detailed explanation of the data.

Clarification of Western Blot Analysis: The reviewer mentioned that UBE2I protein expression in the tumor samples T3, T5, and T6 is similar to that in normal tissues. However, we would like to clarify that while the expression levels in these samples are lower compared to other tumor samples (T1, T2, T4), they are still higher than in normal tissues or adjacent non-tumor tissues. Furthermore, these differences are statistically significant. Therefore, the results are consistent with our hypothesis, which proposed that UBE2I is overexpressed in tumor tissues compared to normal tissues.

Statistical Analysis and Significance: In our calculation of the UBE2I/β-actin ratio, all tumor samples (including T3, T5, T6) showed significantly higher UBE2I protein expression compared to normal or adjacent non-tumor tissues. We have emphasized the statistical significance of these differences in the revised manuscript and ensured that appropriate statistical analyses were conducted for all comparisons between groups.

Tumor Heterogeneity Consideration: We acknowledge that tumor samples exhibit heterogeneity, which can lead to differences in expression levels between individual samples. While some tumor samples (such as T3, T5, T6) have lower UBE2I expression compared to others, they still show higher expression than normal or adjacent tissues. This finding further supports our hypothesis that UBE2I is upregulated in tumor tissues.

**Reviewer #2:**

1. The article mentions the importance of PTC, but it would be useful to expand the discussion on global incidence, survival rates, and the impact of metastatic lymph nodes to emphasize the clinical urgency of new therapeutic targets. More precise details should be provided on the TCGA analysis, including patient selection criteria and the methodology for dividing into high and low UBC9 expression subgroups. Integrating more intuitive graphs to compare survival data between high and low UBC9 groups would enhance visual accessibility.

**Response:** I've revise the manuscript ( Lines 30-33 and 80-83). The graph below compares the survival data between the high and low UBC9 groups. The upper part of the graph shows a scatter plot of gene expression, the horizontal coordinate is the sample, the vertical coordinate is the expression of UBC9, and the colours are used to distinguish the high and low expression groups, and the lower part of the graph shows a scatter plot of survival time and status, the horizontal coordinate is the sample and corresponds to the upper part of the graph, and the vertical coordinate is the overall survival time, and the colours are used to distinguish the survival status.

2. In my opinion, the correlation between UBC9 and the immune microenvironment should be expanded, further exploring the interaction with key immune cells (for example T reg, CD8+). For this purpose, I suggest reviewing this important article, which demonstrates how thyroid hormones together with androgens modulate and amplify the immune microenvironment in neoplastic pathology:https://pubmed.ncbi.nlm.nih.gov/35134514/

**Response:** I've revise the manuscript. ( Lines 460-473).

3. It is necessary to delve deeper into comparisons with other molecules in the SUMOylation pathway already studied in PTC or other cancers. The study's limitations should be discussed, and strategies for future clinical validation proposed.

**Response:** I've revise the manuscript. ( Lines 419-443 and 532-552).

4. Expanding the section on drug sensitivity, specifying which agents might be most promising for patients with high UBC9 expression, would be crucial to strengthen the scientific power of this study.Further exploration is required on how UBC9 could be integrated into combination therapy strategies (for example, immune checkpoint inhibitors or targeted drugs). Drug sensitivity could be explored more thoroughly, specifying which agents might hold the greatest promise for patients with high UBC9 expression. The link between UBC9 and the immune microenvironment could suggest therapeutic combination strategies with immune checkpoint inhibitors or targeted drugs in a tailored therapy regime, as already proposed in other endocrine tumors. For this purpose, I suggest reviewing this article, which highlights how intracellular pathway modulation can lead to the development of intracellular signaling inhibitors for cancer treatment, as demonstrated in prostate cancer, where a correlation was observed between deiodinase upregulation and androgen overproduction: https://pubmed.ncbi.nlm.nih.gov/36930264/

**Response:** I've revise the manuscript. ( Lines 284-295).
